# Supplementary material for: Controlled spin switching in a metallocene molecular junction
Source: Nat Commun. 2017 Dec 7;8:1974. doi: 10.1038/s41467-017-02151-6 (PMC5719446; doi:10.1038/s41467-017-02151-6)
Supplement: Supplementary file 1 — Supplementary Information [file 41467_2017_2151_MOESM1_ESM.pdf]

Supplementary Figures

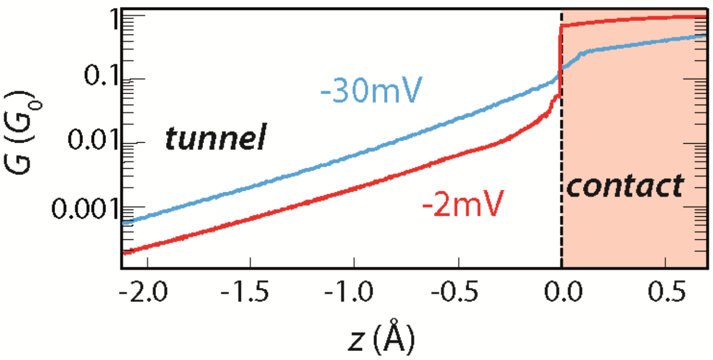

Supplementary Figure 1: Conductance versus displacement in the junction. *G* versus *z* curve at fixed bias -2 mV and -30 mV. At -30 mV there is no abrupt change in conductance as for -2 mV. This is expected since when the bias is increased beyond the excitation threshold the inelastic channel starts contributing to the conductance. Its contribution exceeds by a factor 5 the one from the elastic channel.

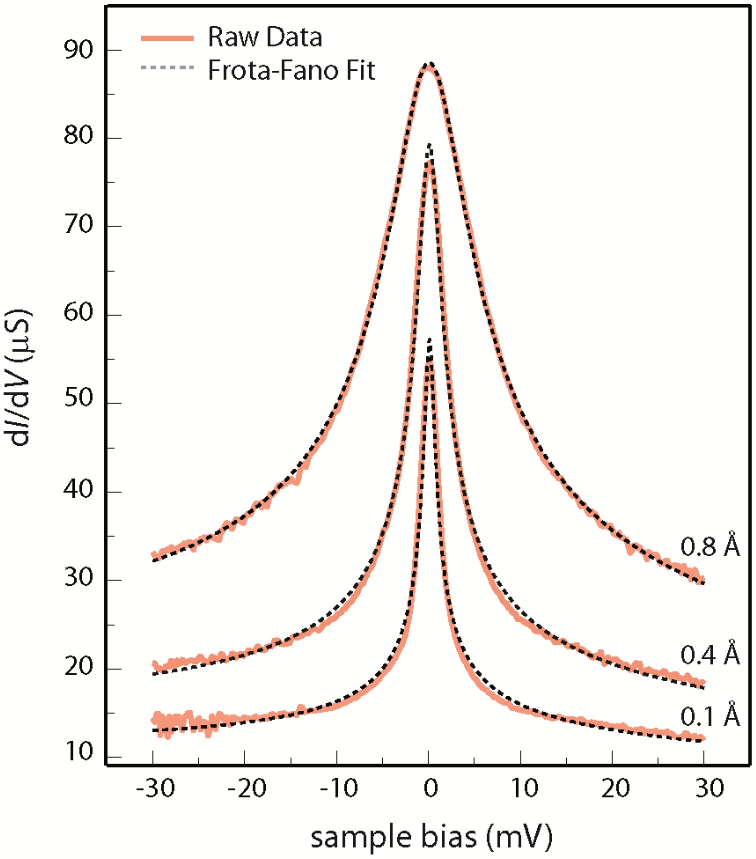

Supplementary Figure 2: Fit of the Kondo resonance. *dI/dV* spectra together with their corresponding Frota-Fano function[1,2] fit are shown for three different Nc-terminated tip-sample distances within the contact regime, z = 0.1, 0.4 and 0.8 **Å**. The obtained fitting parameters are in Supplementary Table 1.

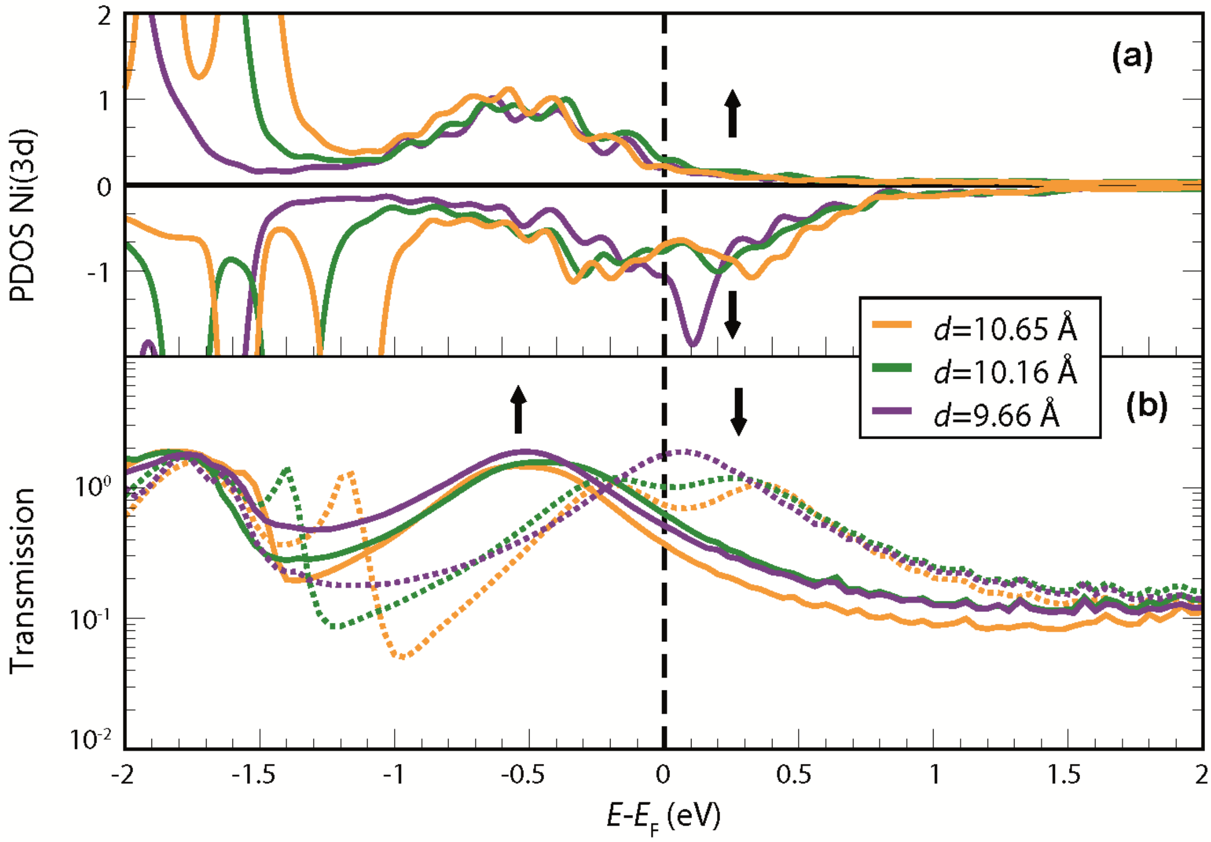

Supplementary Figure 3: Density of states projected (PDOS) and transmission probabilities for majority and minority spins for different configurations in contact. (a) PDOS onto the Ni (3*d*) atomic orbitals for three different configurations in contact. (b) Spin-resolved electron transmission as a function of electron energy with respect to the Fermi energy. As it can be seen, here we have two levels that break their degeneracy (at least partially) and behave in a rather complex way. The levels shift and broaden in a non-monotonous way.

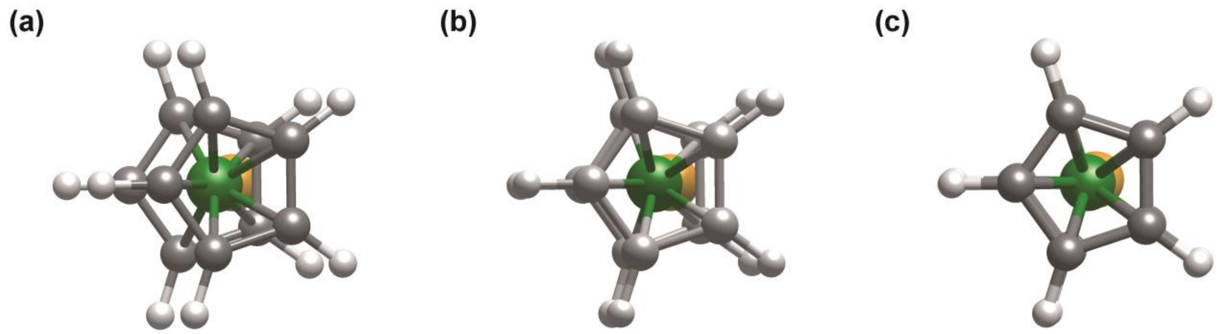

Supplementary Figure 4: Frontal view for the three configurations of interest. (a) The tunneling regime (*d* = 12.17 Å), (b) the transition between the tunneling and contact regimes (*d* = 11.14 Å) and (c) the contact regime (*d* = 9.66 Å). For clarity, the substrate layers have been removed. At contact the Cu adatom is coordinated to the five C atoms of the cyclopentadienyl ring, while in the other configurations it is coordinated to two C atoms.

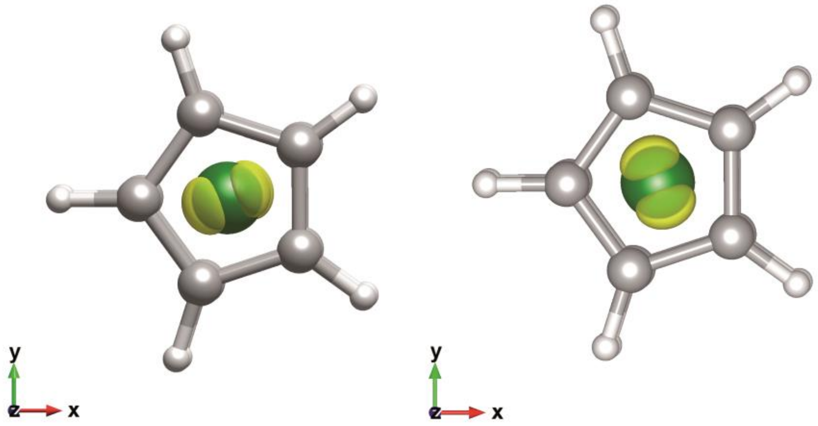

Supplementary Figure 5: Frontier molecular orbitals for the Nc molecule at contact (*d*=9.66 Å). These orbitals have C (2*p*) and Ni (d_{xz}-d_{yz}) contributions but the isovalue was reduced in order to show only the latter contribution.

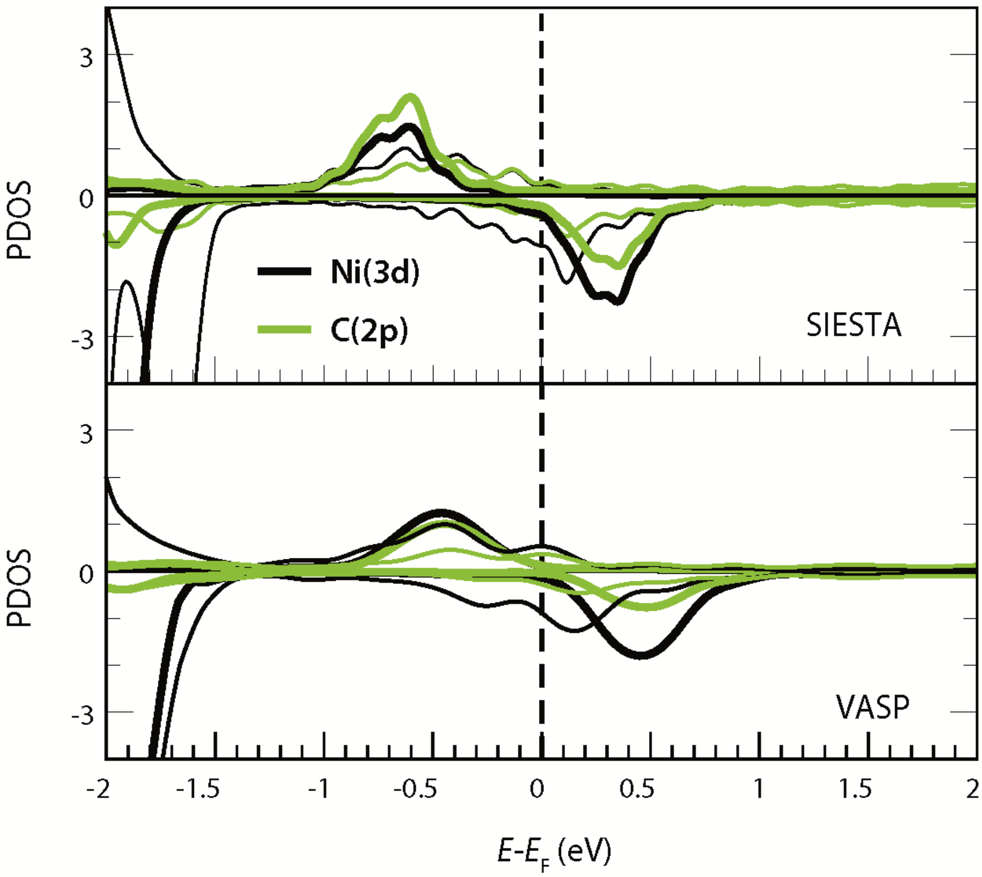

Supplementary Figure 6: Comparison between VASP and SIESTA results. Projected density of states (PDOS) onto C (2*p*) and Ni (3*d*) atomic orbitals as a function of electron energy with respect to the Fermi energy for the Nc-terminated tip / Cu(100) interface. The thick line corresponds to the tunneling (d = 12.17 Å) configuration and the thin line to the contact (d = 9.66 Å) one. A good qualitative agreement is found between both set of results. This information together with the data from charge and magnetization analysis shows that SIESTA and VASP results are describing the same scenario, namely, the reduction of U and increase of Γ as the main factor behind the reduction of the magnetic moment of the molecule in contact. It is worth mentioning that DFT+U calculations with U -J = 2 eV for Ni (3*d*), gives magnetic moments 1.8 μ_B and 1.2 μ_B for the tunnel and contact configurations, respectively.

Supplementary Table

z (Å)	T_K (K)	q	ε_K (meV)
0.1	4.6	>>1	0.1
0.4	9.8	>>1	0.1
0.8	32.7	>>1	0.08

Supplementary Table 1: Kondo fit parameters. Parameters obtained from the fit of the Kondo resonance with a Frota-Fano function. T_K corresponds to the Kondo temperature, q to the Fano parameter and ε_K to the position in which the resonance is centered.

Supplementary References

[1] H. O. Frota, Shape of the Kondo resonance. Phys. Rev. B **45**, 1096-1099, (1992).

[2] H. Prüser, M. Wenderoth, P. E. Dargel, A. Weismann, R. Peters, T. Pruschke and R. G. Ulbrich. Long-range Kondo signature of a single magnetic impurity. Nat. Phys. **7**, 203-206, (2011).
